# Thin Films of Metal-Organic Framework Interfaces Obtained by Laser Evaporation

**DOI:** 10.3390/nano11061367

**Published:** 2021-05-21

**Authors:** Olivia L. Rose, Anca Bonciu, Valentina Marascu, Andreea Matei, Qian Liu, Laurentiu Rusen, Valentina Dinca, Cerasela Zoica Dinu

**Affiliations:** 1 Department of Chemical and Biomedical Engineering, West Virginia University, Morgantown, WV 26506, USA; olrose@mix.wvu.edu (O.L.R.); ql0009@mix.wvu.edu (Q.L.); 2 National Institute for Laser, Plasma and Radiation Physics, RO-077125 Magurele, Romania; anca.bonciu@inflpr.ro (A.B.); valentina.marascu@inflpr.ro (V.M.); andreea.matei@inflpr.ro (A.M.); laurentiu.rusen@inflpr.ro (L.R.); 3 Faculty of Physics, University of Bucharest, RO-077125 Magurele, Romania; 4 IN2—FOTOPLASMAT Center, RO-077125 Magurele, Romania; 5 Université Paris-Saclay, CEA, INRAE, DMTS, SCBM, F-91191 Gif-sur-Yvette, France

**Keywords:** metal-organic frameworks, MOFs, matrix-assisted pulsed laser evaporation, MAPLE, deposited films

## Abstract

Properties such as large surface area, high pore volume, high chemical and thermal stability, and structural flexibility render zeolitic imidazolate frameworks (ZIFs) well-suited materials for gas separation, chemical sensors, and optical and electrical devices. For such applications, film processing is a prerequisite. Herein, matrix-assisted pulsed laser evaporation (MAPLE) was successfully used as a single-step deposition process to fabricate ZIF-8 films. By correlating laser fluency and controlling the specific transfer of lab-synthesized ZIF-8, films with user-controlled physical and chemical properties were obtained. Films’ characteristics were evaluated by scanning electron microscopy (SEM), energy-dispersive X-ray (EDX) spectroscopy, X-ray diffraction (XRD), Fourier transform infrared (FTIR) spectroscopy, and X-ray photoelectron spectroscopy (XPS). The analysis showed that frameworks of ZIF-8 can be deposited successfully and controllably to yield polycrystalline films. The deposited films maintained the integrity of the individual ZIF-8 framework, while undergoing minor crystalline and surface chemistry changes. No significant changes in particle size were observed. Our study demonstrated control over both the MAPLE deposition conditions and the outcome, as well as the suitability of the listed deposition method to create composite architectures that could potentially be used in applications ranging from selective membranes to gas sensors.

## 1. Introduction

Thin films of metal-organic frameworks (MOFs), coordinated combinations of metal ions and organic linkers [1,2], have been proposed for the next generation of user-designed flexible platforms of nanometer porosity to be implemented in a variety of applications ranging from sensors [3,4] to drug delivery [5], and from systems with antimicrobial [6,7] and antibacterial activities [8,9] to selective membranes for gas sensor applications [10,11]. The plethora of MOF applications are supported by the individual particle characteristics, such as high surface area, high pore volume, biocompatibility, and small size [2], with such characteristics being recently controlled through different fabrication methods such as pulsed laser deposition (PLD) [12,13,14], atomic layer deposition (ALD) [15,16], molecular layer deposition (MLD) [17,18], spin coating [19], liquid-phase epitaxy (LPE) [20,21], chemical solution deposition (CSD) [22,23], or Langmuir–Blodgett layer-by-layer (LBL) deposition [24,25], just to name a few. Studies by Fischer et al., for instance, demonstrated that femtosecond PLD led to the formation of zeolitic imidazolate framework (ZIF)-8 thin films; individual ZIF-8 particles were originally synthesized by combining zinc nitrate hexahydrate and 2-methylimidazole. Subsequent soaking of the deposited films in polyethylene glycol 400 (PEG-400) led to changes in their stability [12]. Salmi et al., used ALD to form MOF-5 thin films, with MOF-5 being assembled through the coordination of zinc nitrate hexahydrate and benzenedicarboxylic acid [26] when zinc acetate and 1,4-benzenedicarboxylic acid were used as precursors [15]. Complementarily, Khaletskaya et al., created ZIF-8 thin films by combining ZnO nanolayers fabricated through ALD and confocal radio frequency magnetron sputtering, with the resulting films to be used for applications in microelectronics and miniaturized devices [16], while Lausund et al., combined ALD and MLD to form amino-functionalized UiO-66 thin films for sensor integration and microelectronics [18]. Moreover, LPE allowed the formation of Zn4O(L)3 thin films on Au-coated quartz crystal microbalance (QCM) substrates [20], while CSD (dip coating) implementation led to the formation of ZIF-8 films to be used as vapor sensors [22] or, when combined with polyimide P84, in selective membranes [23]. Lastly, LBL fabrication of NAFS-2 nanofilms on Au or silicon surfaces was proposed for nanodevices [24] or nanotechnological applications [25].

The above listed methods demonstrate the adaptability of the implemented deposition technique to reliably create heterogenous MOF thin films, with the resulting films benefiting from the integration of user-friendly [22] and environmentally friendly [23] synthesis conditions in humid conditions and/or at room temperature, respectively. However, such methods failed to demonstrate resulting films that were uniform in nature [12]; moreover, several of these techniques require long reaction times [15,19] and are not able to provide user-controlled, consistent morphologies [17] or crystallinities [15,17] for the deposited films. Furthermore, PLD, for instance, is known to lead to high degradation of some of the films because of the vapor-related conditions encountered during the film’s growth [12]. With such limitations, the next generation of controlled deposition methods capable of ensuring maximum heterogeneity and continuous, highly oriented film formation, as well as control over their functionality and integrability, is urgently needed. 

Herein, we proposed the use of a gentle laser-based method, i.e., matrix-assisted pulsed laser evaporation (MAPLE), to tailor the formation of films of ZIF-8. Our hypothesis was that MAPLE implementation would also allow controlled deposition in a user-directed manner and at room temperature, thus resulting in films of known crystallinities and uniformities deposited on a selected type of substrate. MAPLE’s versatility was previously demonstrated during the deposition of magnetite silica [27], organic targets [28], colloidal SnO_2_ [29], TiO_2_ nanoparticles [30], kanamycin-functionalized magnetite nanoparticles [31], and polyaniline [32], just to name a few. To demonstrate our hypothesis, we used model MOF ZIF-8, known for its small size [23], biocompatibility, and adaptability [33,34]; its microcrystalline state that is both chemically and thermally stable (up to 500 °C) [35,36]; and its extensive usage in synthetic applications ranging from catalysis to gas separation and sensors [7,8,37]. ZIF-8 was also chosen because previous preparation of thin films from this framework was shown to pose significant difficulties since the bulk material is usually made as brittle crystals or insoluble powders that are not amenable to common surface-processing techniques [38,39]. Above applications are also supported by known N_2_ adsorption data, which showed that ZIF-8′s Brunauer–Emmett–Teller surface area was 1070.7 m^2^/g, while its Langmuir surface area was 1315.6 m^2^/g [40], both in the range of other supporting reports [41].

Our analysis showed that MAPLE deposition can lead to the formation of controlled heterogenous interfaces of ZIF-8 that could potentially be fine-tuned for the multistep deposition of other complex systems or serve in membrane separation applications or as sensors.

## 2. Materials and Methods 

### 2.1. Preparation of ZIF-8 Particles 

**Figure 1:**
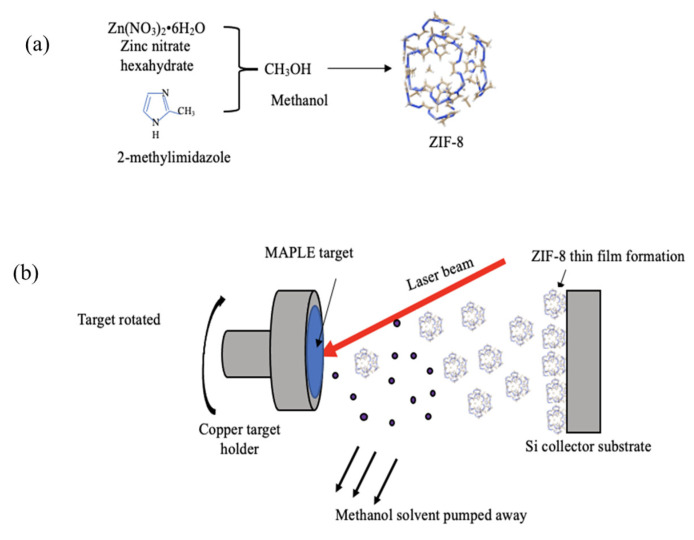
(**a**) Schematic representation of the synthesis process of ZIF-8 MOF particles when using methanol as a solvent. Grey atoms represent the zinc atoms, tan represents the carbon atoms, and white shows the hydrogen atoms. (**b**) Schematic of the controlled deposition of a thin film of ZIF-8 using the MAPLE technique.

ZIF-8 was synthesized by mechanically stirring zinc nitrate hexahydrate (98%, Acros Organics, Fair Lawn, NJ, USA) and 2-methylimidazole (97%, Alfa Aesar, Ward Hill, MA, USA) in methanol solution (HPLC Grade, Fisher Scientific, 99.8% purity, Fair Lawn, NJ, USA), at room temperature, for 24 h. The mole ratio of zinc nitrate hexahydrate/2-methylimidazole/methanol was 1:10:100; the product was collected by centrifugation at 5000 rpm (SORVALL LEGEND X1R Centrifuge, Thermo Scientific, Whaltham, MA, USA) for 5 min and subsequently washed with methanol at least three times (each step followed by centrifugation) to remove any non-precipitated species. The resulting powders were dried at room temperature and stored in a vacuum chamber. Lab-synthesized metal-organic frameworks (MOFs) of ZIF-8 are shown in Figure 1a.

### 2.2. Target Preparation

Targets of ZIF-8 were formed by suspending the lab-synthesized MOFs in a methanol solution to lead to a concentration of 0.5 wt.%; the resulting mixture was subsequently frozen in a copper holder at around −196 °C in liquid nitrogen. Targets were immediately used for the laser deposition process. 

### 2.3. Matrix-Assisted Pulsed Laser Evaporation (MAPLE) for Film Formation

A Nd:YAG (Surelite II pulsed laser system, Continuum Company, Pessac, France) pulsed laser system operating at 266 nm, with a 6 ns pulse duration and under a 10 Hz frequency, was used to irradiate the surface of a pre-formed target. The laser beam spot size was maintained at 1.5–2 mm; the size of the beam was measured by placing thermally sensitive paper in the plane of the target, at an incidence angle of 45°. For uniform target evaporation, the laser beam was translated onto the target surface while the target was rotated using the motion feed of an integrated motor. The combined translation–rotation motions were used to avoid excessive target heating, also known to lead to target erosion [42]. The conversion of beam energy into thermal energy [28] at the target’s interface led to a gentle film transfer onto a Si collector substrate (IR transparent single-side-polished silicon wafer) placed at a distance of 4 cm.

The consideration for the 4 cm controlled distance was based on previous studies that showed that if smaller distances are to be considered, possible clustering of material on the target surface could occur, thus challenging film quality. The mechanism of such clustering was discussed by Shepard et al., [43] and is based on the deflation of bubbles containing clusters onto the receiver target. Methanol solvent resulting upon MAPLE deposition was pumped away from the vacuum chamber (Figure 1b). The number of pulses used in all the experiments was 18 k pulses, while the laser fluence varied between 0.3 and 0.8 J∙cm^−2^. All depositions took place in vacuum (1 × 10^−4^ mbar) maintained using a “PfeifferBalzers TPU 170” (Albuquerque, NM, USA) turbomolecular pump (170 L s^−1^ volume flow rate).

### 2.4. Characterization of the MAPLE Deposited Films and of Controls of ZIF-8 

Physical and elemental composition analysis of the lab-synthesized ZIF-8 and MAPLE deposited films was performed using a JSM-531Inspect S SEM (Hillsboro, OR, USA) at accelerating voltages between 20 and 25 kV. An EDX (Element 2CB) attached to an FEI Inspect S SEM operating at 10 keV was used to characterize the elemental composition of the target and the obtained films qualitatively and quantitatively; at least three runs were compiled. Statistical accuracy was increased by using the OriginLab (Northampton, MA, USA) compatible software platform, with the scale bar subsequently traced and set for all the imported images.

For chemical characterization, Fourier transform infrared (FTIR) spectroscopy and X-ray photoelectron spectroscopy (XPS) were performed. For the first, a Jasco 6300 FTIR system (Oklahoma, OK, USA) spectrometer operating in transmission mode, with a range of 400–4000 cm^−1^, and at a resolution of 4 cm^−1^ was used. The infrared spectrum of the native material (i.e., lab-synthesized ZIF-8) drop cast on the Si substrate (IR transparent single-side-polished silicon wafer) was used as a reference. For XPS (Thermo Scientific, Waltham, MA, USA) survey spectra and high-resolution XPS scan spectra, drop-cast films (controls) and films obtained by MAPLE were used. The spectra were acquired using an Al Kα gun with a spot size of 900 µm, a pass energy of 100.0 eV, and an energy step size of 1.00 eV. For the high-resolution XPS spectra, the pass energy was set to 10.0 eV and the energy step size was 0.10 eV, with 10 scans accumulated and averaged.

Lastly, X-ray diffraction (XRD), performed using a PANalytical X’Pert MPD diffractometer (Almelo, The Netherlands) system operating at a λ of 0.15418 nm, was used to investigate the crystal structures of both the starting material and the resulting, deposited films.

## 3. Results and Discussion

Next-generation thin films formed from lab-synthesized metal-organic frameworks (MOFs) were obtained by MAPLE (Figure 1) at different laser fluencies. The deposited films’ physical and chemical characteristics were investigated using scanning electron microscopy (SEM), Fourier transform infrared (FTIR) spectroscopy, X-ray diffraction (XRD), energy-dispersive X-ray spectroscopy (EDS), and X-Ray photoelectron spectroscopy (XPS). 

### 3.1. Morphological Analysis of Deposited Samples Relative to Controls 

**Figure 2:**
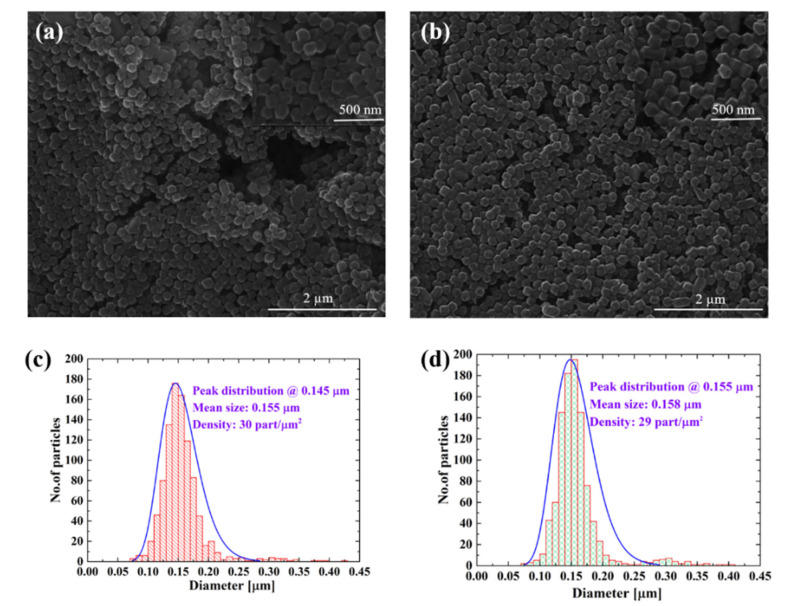
(**a**) SEM image of representative ZIF-8 control with inset showing a higher-resolution analysis of individual ZIF-8 constituents; (**b**) SEM image of representative ZIF-8 film deposited by MAPLE at a 0.45 J∙cm^−2^ fluence, with inset presenting a higher-resolution analysis of the deposits; (**c**) Histogram of ZIF-8 control with lognormal fit function (blue line); (**d**) Histogram of ZIF-8 thin film deposited via MAPLE using a 0.45 J∙cm^−2^ fluency with lognormal fit function (blue line).

SEM analysis showed that MAPLE deposition using different laser fluences (i.e., 0.3, 0.45, 0.6, and 0.8 J∙cm^−2^) generally led to a uniform distribution of ZIF-8 particles on Si substrates. The analysis also showed that deposited particles maintained their integrity and did not significantly change their shape or size when compared to controls (Figure 2a,b, respectively). Additional SEM morphology analysis results for samples deposited at 0.3, 0.6, and 0.8 J∙cm^−2^ are included in Figure S1.

The statistical size distributions of the rhombic dodecahedron morphologies measured over a 34.6 µm^2^ surface area are shown in Figure 2c for ZIF controls and in Figure 2d for a MAPLE sample deposited at 0.45 J∙cm^−2^. The recorded peak distribution for control ZIF-8 was at 0.145 µm, with mean particle diameter of 0.155 µm ± 0.03 µm and particle density of 30 part/µm^2^. For the MAPLE sample deposited at a fluence of 0.45 J∙cm^−2^, the histogram revealed a peak distribution at 0.155 µm, a mean particle diameter of 0.158 µm ± 0.03 µm, and particle density on the substrate of 29 part/µm^2^. These analyses showed that while the mean diameters of both investigated materials (control and deposited via MAPLE) were rather similar, the recorded peak distributions seemed to have changed slightly. Specifically, the histogram showed a predominance of particles (667 particles) with sizes between 0.135 and 0.165 µm for the MAPLE deposited films; for the control, there were only 594 particles of this size. Moreover, the analysis also seemed to indicate the appearance of a double histogram at values between 0.250 and 0.350 µm for the MAPLE films. Samples deposited at fluences of 0.3, 0.6, and 0.8 J∙cm^−2^ also revealed similar particle diameters, as well as peak distributions with a similar value range (0.145–0.165 µm; Figure S2) with two histogram distributions.

The small size changes recorded between samples deposited using different laser fluencies could presumably be due to MOF melting and/or surface evaporation [44,45] upon laser interaction with lab-synthesized ZIF-8. In particular, absorption of the laser’s photons by the particle’s electrons is known to lead to an accumulation of surface energy, with additional transfer of such energy to the sample’s crystal lattice [46]. Moreover, the laser energy absorbed by the target formed from individual ZIF-8 is converted into thermal energy that could potentially result in local heating and evaporation [28,47] of the constituents. This is supported by Lock et al., who showed how localized heating on the surface of lab-synthesized MOFs could lead to negative thermal expansion (contraction) of MOF-5 [48]. Lastly, freezing the target in liquid N could also lead to changes in the constituent sizes; this is supported by previous studies by Wee et al., who demonstrated that Cu_3_(BTC)_2_ particle sizes with initial diameters of 200–300 nm shrank to about 100 nm after freezing in liquid nitrogen (−196 °C) or after freeze drying (−60 °C) [49]. 

Changes in thickness were also investigated; SEM cross sections showed that small changes occurred and were dependent on the fluence used (Supplementary Materials, Figure S3). The observed changes based on fluence were due to ZIF-8 material being ejected from the target via a mechanism previously reported by Shepard et al., [4]; the authors noted small variations in height resulting from the deflation of bubbles containing material to be deposited on the given substrate.

### 3.2. Chemical and Structural Analyses of the Deposited Samples Relative to Controls 

**Figure 3:**
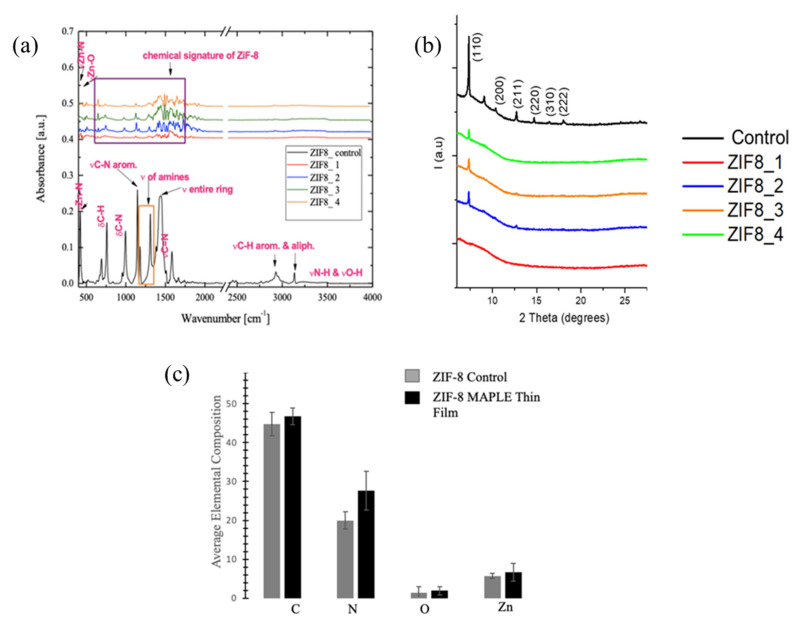
(**a**) FTIR spectra of ZIF-8 control and representative MAPLE deposited films (obtained at fluences of 0.3 J cm^−2^ (ZIF8_1), 0.45 J cm^−2^ (ZIF8_2), 0.6 J cm^−2^ (ZIF8_3), and 0.8 J cm^−2^ (ZIF8_4)). (**b**) XRD analysis of ZIF-8 control and MAPLE samples deposited at different laser fluencies; (**c**) Respective average elemental compositions of ZIF-8 control and representative MAPLE deposited samples.

FTIR (Figure 3a) analysis of lab-synthesized ZIF-8 revealed peaks in the range 3200–3600 cm^−1^ corresponding to νN–H and νO–H [50,51] groups; peaks at 2928 cm^−1^ and 3134 cm^−1^ corresponding to the νC–H aromatic and aliphatic stretching of the imidazole unit [50,51,52]; and a peak at 1584 cm^−1^ corresponding to the νC=N stretching vibration. Stretching vibration of the entire imidazole ring was also recorded at 1344–1500 cm^−1^ [51,52], while amine stretching vibrations were observed in the range 1170–1340 cm^−1^ and at 1145 cm^−1^ and were attributed to νC–N [53]. The wavenumbers 994 and 757 cm^−1^ were associated with the bending vibrations of δC–N and δC–H, while the stretching vibration of Zn-N occurred at 422 cm^−1^ [50,51,52]. Similarly, the MAPLE deposited films at different laser fluences (i.e., 0.3, 0.45, 0.6, and 0.8 J∙cm^−2^) showed the chemical signature of lab-synthesized ZIF-8 (control) in the 620–1640 cm^−1^ range, with no significant variations between these samples. Moreover, νZn–O stretching vibration occurred around 450 cm^−1^ [53]. In the 1257 to 1640 cm^−1^ region, however, the absorption of the deposited films seemed to be combined with the absorptions of the atmospheric O-H, while in the 2928–3135 cm^−1^ range the absorptions of νC–H aromatic and aliphatic stretching vibrations of the imidazole were missing. The additional peaks recorded at 2928 cm^−1^ and 3134 cm^−1^ were also missing, with such peaks previously attributed to the νC–H aromatic and aliphatic stretching of the imidazole unit [50,51,52].

The lack of specific peaks in the deposited thin film is presumably due to changes induced upon laser radiation and resulting laser energy adsorption by the target, with the small variations being a function of the fluency used. Specifically, imidazole units were previously shown to undergo decomposition following electronic state excitation when using pulsed laser irradiation [54], with such decomposition leading to additional rotations/vibrations, as well as π*←n transitions, for most nitro-containing model molecules, as well as possible decay processes induced by the attachment/changes in a single electron-related transfer process [55]. This is supported by previous analysis by Grumezescu et al., which demonstrated that transfer of kanamycin-functionalized magnetite nanoparticle thin films at a laser fluence of 0.5 J∙cm^−2^ could lead to significant disruptions of the main functional groups of the compound [31]; moreover, others demonstrated impairment of the imidazolate functional group of ZIF-8 upon decomposition of the material [28].

XRD (Figure 3b) showed that the deposited ZIF-8 films displayed only one characteristic peak of the lab-synthesized ZIF-8 control, namely, at around 2θ = 6° corresponding to the (110) plane [50,53]. All the other characteristic peaks of the ZIF-8 control (i.e., those at around 2θ = 11°, 12.5°, 15°, 16.5°, and 17.5°, corresponding to planes (200), (211), (220), (310), and (222), respectively [34,53]) were missing for all used fluencies. The observed changes in the individual sample crystallographic structures were presumably due to changes induced by the target-absorbed laser energy. Specifically, such absorption could lead to an accumulation of surface energy onto an individual lab-synthesized MOF with subsequent transfer to the sample’s crystal lattice, resulting in local decomposition through melting and/or subsequent evaporation [46]. Indeed, previous analysis by Lancok et al., showed that, for instance, Eu^3+^-doped yttrium oxide films prepared by laser deposition undergo local temperature changes that induce crystalline defects [56]. Further, Suda et al., showed that nitrogen-doped titanium oxide thin films formed through laser deposition could be influenced by the deposition conditions, with the authors observing that when the ratio of N_2_:O_2_ in the deposition chamber was 1:0, for instance, the (101) peak disappeared from the resulting film [57]. Lastly, the individual placement of the ZIF-8 rhombic dodecahedron planes in the path of the laser beam can also result in certain planes absorbing more energy relative to their neighboring ones, thus leading to additional defects in the resulting films^56^. This is further supported by Lock et al., who demonstrated how localized heating on the surface of selected MOFs could lead to negative thermal expansion and framework cell parameter changes [48].

The samples’ elemental compositions as recorded by EDS also confirmed that while all samples contained C, N, O, and Zn (Figure 3c), consistent with ZIF-8′s imidazolate linker and metal ion, zinc [33], there were slight increases in C, N, and Zn recorded for the MAPLE deposited samples, additionally supporting slight decomposition of the starting material [12,28] (Table S1).

**Figure 4:**
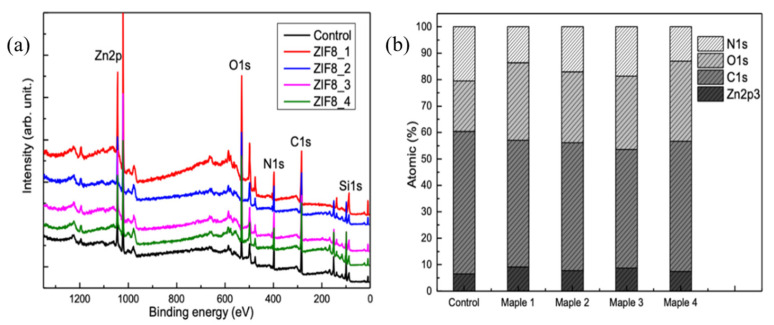
XPS survey spectra of ZIF-8 control and MAPLE deposited films presented using both changes in intensity (**a**), as well as atomic ratios (**b**).

XPS investigations supported changes in the films’ characteristics, with such changes being a function of the laser fluency used during the MAPLE deposition process. Specifically, Figure 4 presents the characteristics peaks—namely, those of C1s, N1s, O1s, and Zn2p—found in the survey spectra of both ZIF-8 control and MAPLE deposited samples. The Si1s peak from the deposition substrate was also present. The elemental distribution is included in Supplementary Materials, Table S2, with the specific binding energies obtained using C1s at 284.8 eV for calibration.

Figure S4 contains the high-resolution spectra, N1s, C1s, and O1s, while Figure S5 contains the Zn2p high-resolution XPS spectra of the ZIF-8 control and MAPLE deposited ZIF-8 film obtained at 0.45 J∙cm^−2^. Specifically, while the ZIF-8 control showed the characteristic Zn2p3 peak at 1022.92 eV [58], the main peak of O1s at 533.52 eV was assigned to the carbonates [59]; for the N1s peak of the ZIF-8 control and MAPLE deposited samples, the spectrum was fitted with two peaks corresponding to C–N and C=N in imidazole [59,60] at about 398.2 and 399.3 eV, while the C1s peak at 285.23 eV corresponded to the 2-methylimidazole of the ZIF-8 [58,59]. The peaks for the deposited ZIF-8 MAPLE thin film were slightly shifted towards lower energies, i.e., 1021.03, 531.19, 398.42, and 284.34 eV, respectively. The C1s spectrum was deconvoluted in four peaks, attributed to C–C, C–N, C–O or C–N, and C–OH bonds [61]. Traces of methanol were also identified for the MAPLE samples. The O1s spectrum was deconvoluted in three peaks assigned to Zn–O, O–C, and O–H/H_2_O [59]. Further, the elements Zn and O were increased in intensity in the MAPLE deposited thin film when compared to the control, while N and C decreased in intensity again relative to the control lab-synthesized ZIF-8. 

The above results support changes in the MAPLE deposited samples relative to controls, with such changes being a function of the laser fluency used. The observed changes were presumably due to laser adsorption during the MAPLE deposition process, with such adsorption leading either to decomposition of the starting material or surface changes [28]. These are supported by Pique et al., who showed that organic thin films obtained through MAPLE at laser fluence ranging from 0.01 to 0.5 J∙cm^−2^ showed changes of the polymer, mostly due to decomposition [28]. In addition, research by Wang et al., showed that polyimide thin films deposited by MAPLE presented starting material decomposition when the laser fluence decreased to 0.5 J∙cm^−2^ [62]. Nevertheless, for all the deposited films, the surface was Zn enriched when compared to the starting control and its imidazole content, like in the experiments conducted by Fangyuan Tian et al., thus demonstrating partial decomposition of ZIF-8 as also shown through XRD and FTIR analysis [59]. However, the Zn/N atomic ratio showed that films obtained at 0.45 and 0.6 J∙cm^−2^ (i.e., ZIF8-2 and ZIF8-3) maintained the closest chemical structure to the control sample, thus demonstrating that these two fluencies are the ones that best preserve the characteristics of the starting material, i.e., lab-synthesized ZIF-8. 

The observed results demonstrate MAPLE feasibility, as well as user ability to control deposition characteristics by controlling the laser fluency used during the deposition process. We envision that controlled deposition of MOF films using MAPLE could be tuned to create structures with applications in sensors or membranes [63] or to form seeding layers [64] in which such deposited films serve as nucleation centers for the growth of catalytic precursors. Such tuning capability combined with the ability to deposit on a variety of substrates, from metals, to oxides, to polymers, all at room temperature, could allow for increased versatility of deposited thin films while largely maintaining their physicochemical characteristics.

## 4. Conclusions

The MAPLE technique was used to form ZIF-8 films on Si substrates; analysis of the films’ physical and chemical properties showed no significant changes in their morphology, shape, or size. However, their chemical and structural characterization showed that slight surface changes and decomposition of the starting material occurred upon transfer at different laser fluencies, with such processes being presumably due to the laser absorption at the individual MOF interface, known to occur during the MAPLE deposition process. Herein, the demonstrated ZIF-8 structure transfer by laser evaporation is seen to allow for controlled film deposition to help dictate film functionality and integrability when practical control of its parameters and deposition is permitted (i.e., at different fluencies or numbers of pulses).

## Data Availability

The data presented in this study is available on request from the authors.

## Supplementary Materials

The following information is available online at https://www.mdpi.com/article/10.3390/nano11061367/s1, Figure S1: SEM analysis of MAPLE deposited film samples at different laser fluences, Figure S2: Histogram size analysis of MAPLE deposited samples at different laser fluencies, Figure S3: Cross sections of thin films; Figure S4: C1s, O1s, and N1s high-resolution XPS spectra of ZIF-8 control and MAPLE deposited ZIF-8 films obtained at 0.45 J∙cm−2 fluency, Figure S5: Zn2p high-resolution XPS spectra of ZIF-8 controls and MAPLE deposited ZIF-8 thin films, Table S1: Element average weight percent of MAPLE films obtained at different fluencies, Table S2: Atomic ratios for ZIF-8 control and MAPLE deposited ZIF-8 films. The Zn doublet Zn 2p3/2 and Zn 2p1/2 can be identified at 1022.8 eV and 1046 eV, with the characteristic split spin-orbit components (23.2 eV).
