# Supplementary material for: Thin Films of Metal-Organic Framework Interfaces Obtained by Laser Evaporation
Source: Nanomaterials (Basel). 2021 May 21;11(6):1367. doi: 10.3390/nano11061367 (PMC8224316; doi:10.3390/nano11061367)
Supplement: Supplementary file 1 [file nanomaterials-11-01367-s001.zip › nanomaterials-1169438-supplementary.pdf]

Supplementary Materials

Thin films of metal-organic frameworks interfaces obtained by laser evaporation

Olivia L. Rose [1], Anca Bonciu [2,£,3], Valentina Marascu[2,4], Andreea Matei[2], Qian Liu[1], Laurentiu Rusen[2], Valentina Dinca [2,£,] and Cerasela Zoica Dinu [1,*]**

[1] Department of Chemical and Biomedical Engineering, West Virginia University, Morgantown, WV 26506 USA; cerasela-zoica.dinu@mail.wvu.edu

[2] National Institute for Laser, Plasma and Radiation Physics, Magurele, Ilfov, RO-077125, Romania

[£] IN2 - FOTOPLASMAT center, Magurele, RO-077125 Ilfov, Romania

[3] Faculty of Physics, University of Bucharest, Magurele, RO-077125 Ilfov, Romania; valentina.dinca@inflpr.ro

[4] Université Paris-Saclay, CEA, INRAE, DMTS, SCBM, F-91191 Gif-sur-Yvette, France

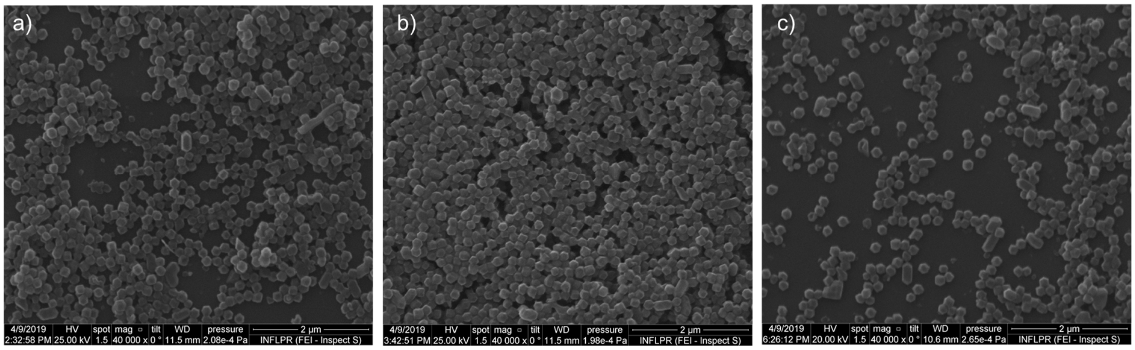

Figure 1. SEM images of MAPLE samples deposited at different fluencies, i.e., 0.3 (a); 0.6 (b) and 0.8 J·cm^{-2} (c) respectively.

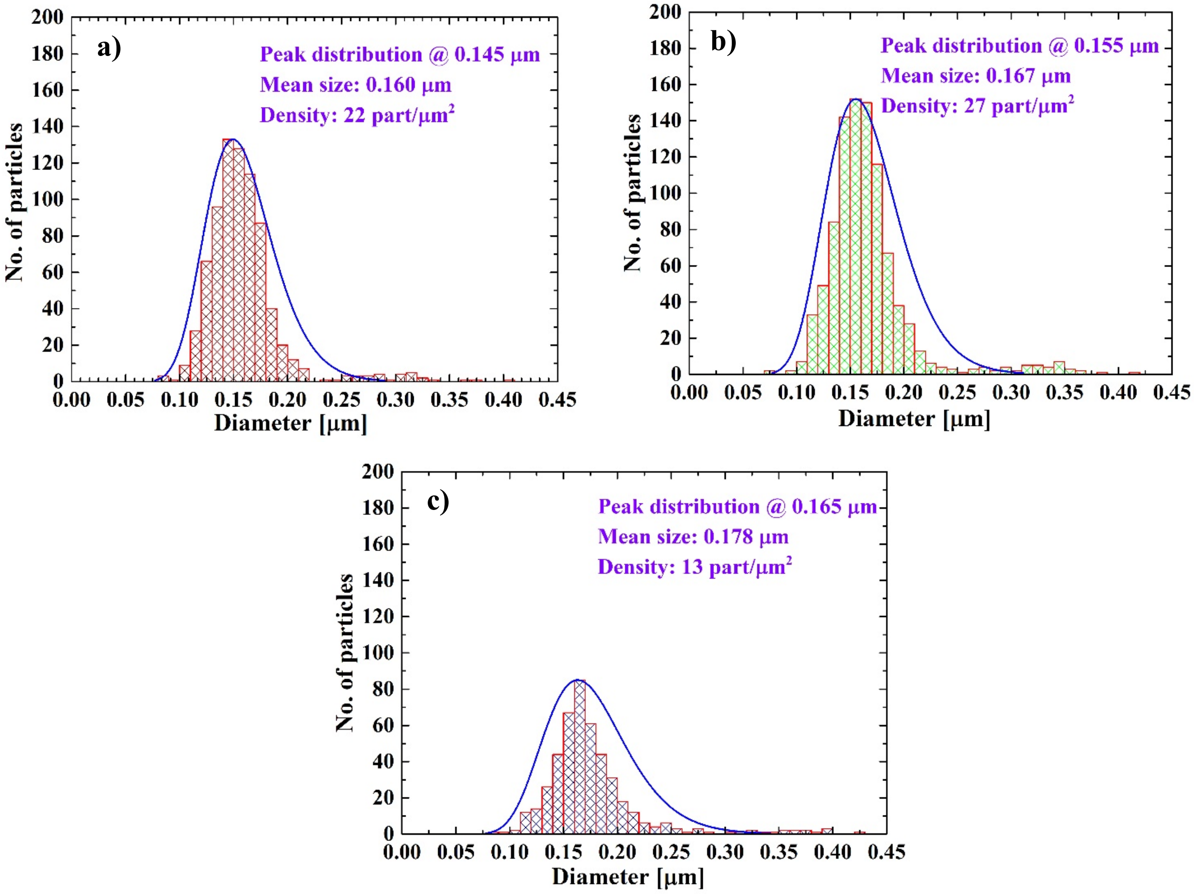

Figure S2: Histogram size analysis of MAPLE deposited samples at different laser fluencies (a for 0.3 $J \cdot cm^{-2}$, b for 0.6 $J \cdot cm^{-2}$ and c for 0.8 $J \cdot cm^{-2}$ respectively)

Table S1. Element average weight percent of MAPLE films obtained at different fluencies.

Element Average Weight percent (%)	Control ZIF8	ZIF8 (0.300 J·cm^{-2})	ZIF8 (0.450 J·cm^{-2})	ZIF8 (0.600 J·cm^{-2})	ZIF8 (0.800 J·cm^{-2})
C	44.74 ± 3.04	16.07 ± 12.76	47.27 ± 0.99	46.70 ± 2.12	32.18 ± 8.52
N	19.97 ± 2.12	3.65 ± 4.04	29.75 ± 3.01	27.59 ± 5.01	10.73 ± 5.00
O	1.44 ± 1.59	0.57 ± 0.48	1.81 ± 0.53	1.94 ± 1.05	0.47 ± 0.40
Zn	5.73 ± 0.69	1.52 ± 0.86	7.43 ± 1.65	6.69 ± 2.25	1.97 ± 0.66

Table S2. Atomic ratios for ZIF-8 control and MAPLE deposited ZIF-8 films.
The Zn doublet Zn 2p3/2 and Zn 2p1/2 can be identified at 1022.8 eV and 1046 eV, with the characteristic split spin-orbit components (23.2 eV).

	ZIF8 Control	ZIF8 (0.300 J·cm^{-2})	ZIF8 (0.450 J·cm^{-2})	ZIF8 (0.600 J·cm^{-2})	ZIF8 (0.800 J·cm^{-2})
	**Atomic %**	**Atomic %**	**Atomic %**	**Atomic %**	**Atomic %**
**Zn2p3**	6.52	9.17	7.73	8.7	7.42
**C1s**	53.91	47.82	48.44	44.88	49.37
**O1s**	19.03	29.39	26.78	27.75	30.21
**N1s**	20	13.92	17.05	18.67	13
**Zn2p3/N1s**	0.317	0.673	0.453	0.466	0.570

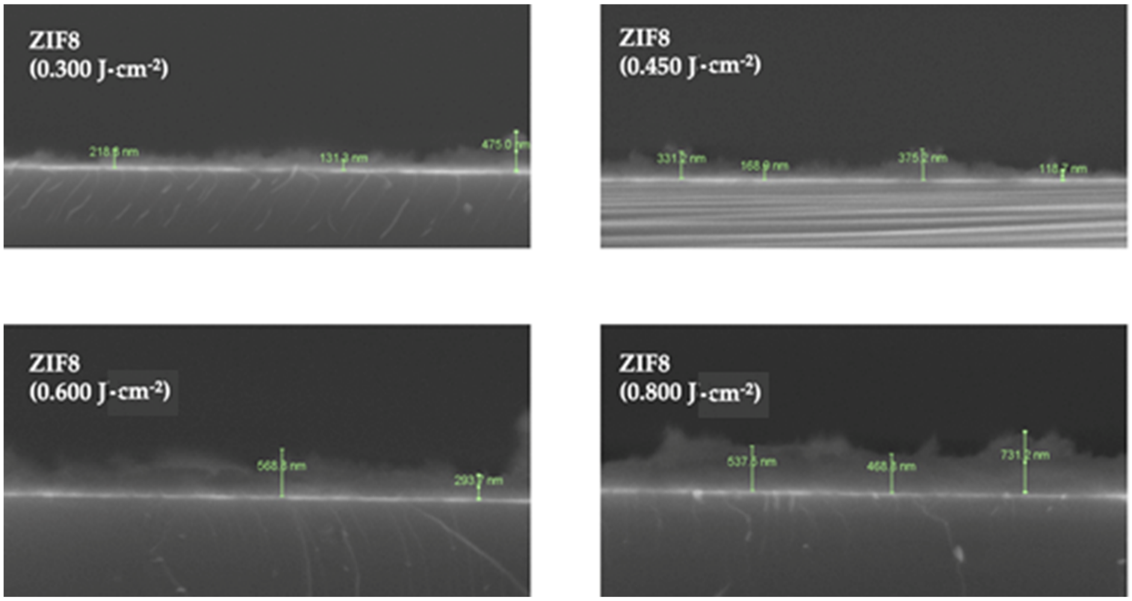

Figure S3: Cross-section and thin film thickness dependence on the frequency being used.

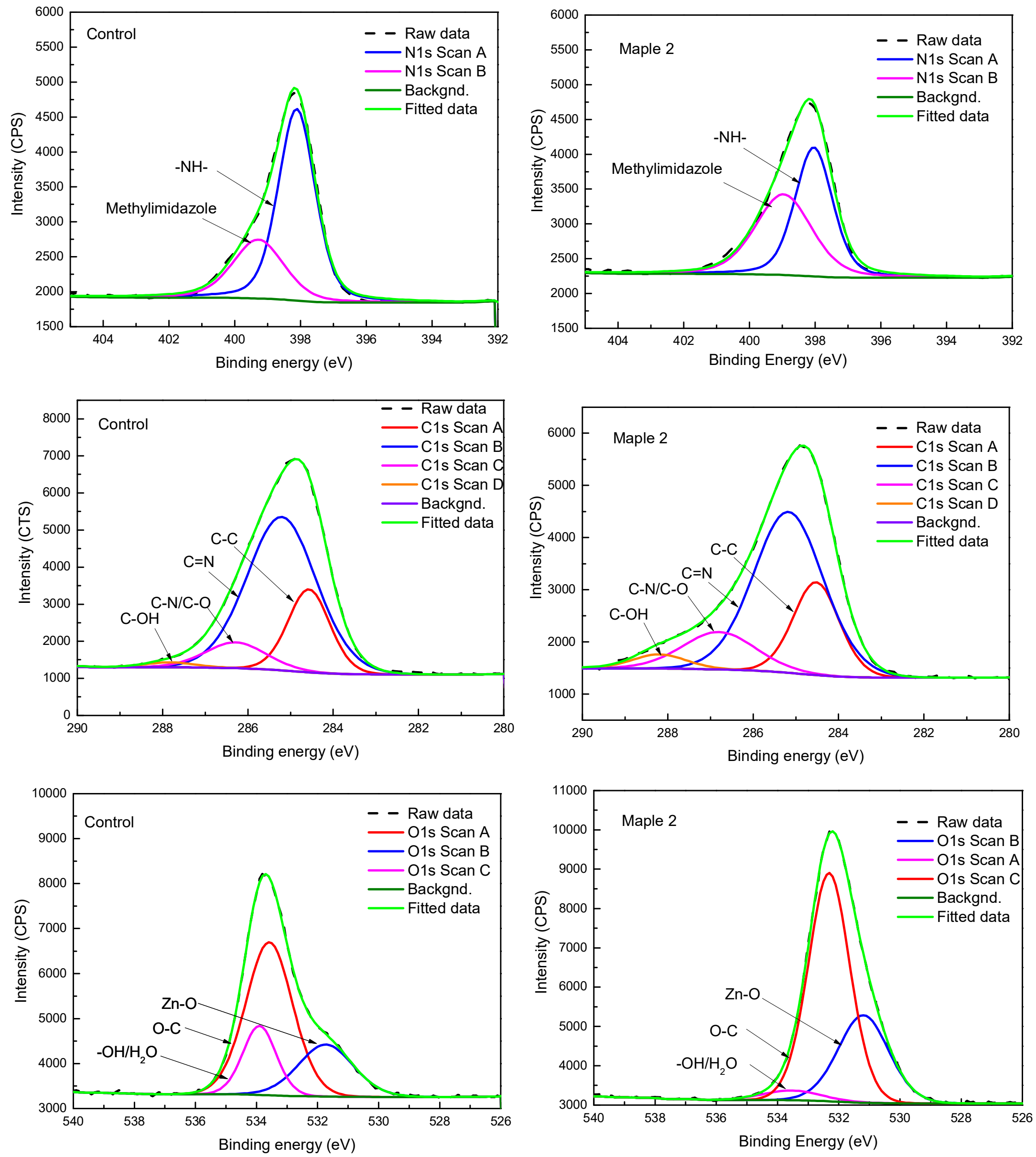

Figure S4. C1s, O1s and N1s high-resolution XPS spectra of ZIF-8 control and MAPLE deposited ZIF-8 film obtained at the 0.45 Jcm^{-2} fluency.

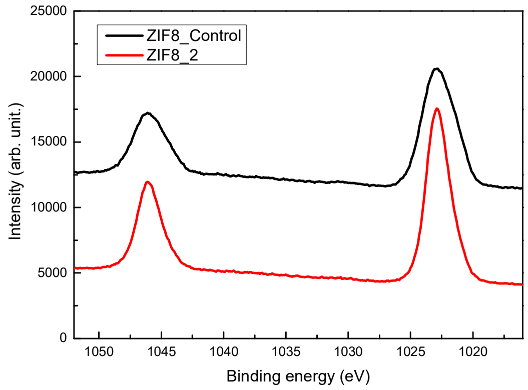

Figure S5: Zn2p high-resolution XPS spectra of ZIF-8 control and MAPLE deposited ZIF-8 thin film at 0.450 J·cm^{-2}.
